# The Tripartite Rhizobacteria-AM Fungal-Host Plant Relationship in Winter Wheat: Impact of Multi-Species Inoculation, Tillage Regime and Naturally Occurring Rhizobacteria Species

**DOI:** 10.3390/plants10071357

**Published:** 2021-07-02

**Authors:** Thomas I. Wilkes, Douglas J. Warner, Veronica Edmonds-Brown, Keith G. Davies, Ian Denholm

**Affiliations:** 1 Department of Psychology, Sport and Geography, School of Life and Medical Sciences, College Lane Campus, University of Hertfordshire, Hatfield, Hertfordshire AL10 9AB, UK; v.r.edmonds-brown@herts.ac.uk (V.E.-B.); k.davies@herts.ac.uk (K.G.D.); i.denholm@herts.ac.uk (I.D.); 2 Agriculture and Environment Research Unit, School of Life and Medical Sciences, College Lane Campus, University of Hertfordshire, Hatfield, Hertfordshire AL10 9AB, UK; d.j.warner@herts.ac.uk

**Keywords:** arbuscular mycorrhizal fungi, plant growth promoting rhizobacteria, wheat, soil inoculum, multi-species interactions, *Bacillus amlyoliquefaciens*

## Abstract

Soils and plant root rhizospheres have diverse microorganism profiles. Components of this naturally occurring microbiome, arbuscular mycorrhizal (AM) fungi and plant growth promoting rhizobacteria (PGPR), may be beneficial to plant growth. Supplementary application to host plants of AM fungi and PGPR either as single species or multiple species inoculants has the potential to enhance this symbiotic relationship further. Single species interactions have been described; the nature of multi-species tripartite relationships between AM fungi, PGPR and the host plant require further scrutiny. The impact of select *Bacilli* spp. rhizobacteria and the AM fungus *Rhizophagus intraradices* as both single and combined inoculations (PGPR_[i]_ and AMF_[i]_) within field extracted arable soils of two tillage treatments, conventional soil inversion (CT) and zero tillage (ZT) at winter wheat growth stages GS30 and GS39 have been conducted. The naturally occurring soil borne species (PGPR_[s]_ and AMF_[s]_) have been determined by qPCR analysis. Significant differences (*p* < 0.05) were evident between inocula treatments and the method of seedbed preparation. A positive impact on wheat plant growth was noted for *B. amyloliquefaciens* applied as both a single inoculant (PGPR_[i]_) and in combination with *R. intraradices* (PGPR_[i]_ + AMF_[i]_); however, the two treatments did not differ significantly from each other. The findings are discussed in the context of the inocula applied and the naturally occurring soil borne PGPR_[s]_ present in the field extracted soil under each method of tillage.

## 1. Introduction

Microbial associations between plant and soil are highly complex and provide a myriad of interactions with a wide range of plant and soil benefits including increased soil fertility and aggregation [1,2], improved plant immunity and defence [3], increased plant biomass and carbon sequestration [4]. Arbuscular mycorrhizal (AM) fungi are one of the constituent organisms within the rhizosphere of an estimated 80% of terrestrial plants [1] forming mutualistic biotrophic symbiosis with host plants [5]. Current predictions suggest a historical relationship dating back 400 million years [6]. Within an agricultural setting, this can be of commercial importance for the amplified nutrient availability produced from AM fungal acquired soil bound nutrients, such as phosphorus, improving crop health, biomass and yield [7]. AM fungi can additionally provide benefits to the improvement of soil properties, such as carbon and aggregation [2,8]. However, in combination with Plant Growth Promoting Rhizobacteria (PGPR), soil fertility can be improved further [9]. Mycorrhizal helper bacteria (MHB) stimulate mycorrhizae formation and further enhance plant-fungi symbiosis [10]. The role of MHB and their interaction with AM fungi is poorly understood as is the extent of the tripartite rhizobacteria-AM fungi-host plant relationship.

Within the soil rhizosphere, many microbial species interact with each other, and the nature of this interaction may be both beneficial and detrimental to crop growth. An increase in extra radial AM fungal mycelia may be a direct result of MHB associations [11], while an increase in phosphorus uptake from soil bound sources, via AM fungi, are indirect [12]. Bacterial taxa such as *Firmicutes*, *Oxalobacteraceae* and *Actinobacteria* have been shown to exist in proximity to extra radiating fungal mycelia and aid in the continued proliferation of mycelial networks [13,14]. Further, *Bacillus subtilis* (MB1600 and GB122) and *B. amyloliquefaciens* (BG99) are reported by Borriss [15] to have direct plant growth promoting properties. Infection by pathogenic bacteria and fungi, such as genera *Xanthomonas* and *Fusarium*, may also be inhibited by PGPR via a direct biocontrol effect or indirectly through competition for resources [16]. The nature of the tripartite relationship is multi-faceted and consequently may not always be beneficial. *Bacillus subtilis* for example has been shown to inhibit the AM fungi *Rhizophagus intraradices* when applied as an inoculant of winter wheat [17] despite being reported as having a positive interaction with AM fungi by Alam et al. [18]. It is evident that a multitrophic community exists with complex interactions between the plant, PGPR and AM fungi. Perez-de-Luque et al. [19] suggest plant species and crop variety are one such factor. Crop management interventions, for example tillage regime, may be another.

Soil cultivation by conventional tillage (CT) has been shown to directly influence the abundance and diversity of the soil microbiome [20]. The hyphal networks of AM fungi are damaged by CT [21] resulting in decreased root cortical arbuscules [2,22]. The impact of tillage on rhizobacteria is relatively unknown. A tillage system reported to be detrimental to AM fungi development, i.e., CT [2,20,21,22,23], may benefit from inoculation with AM fungi. This benefit however may not be realised until later crop growth development stages. Further, this benefit may be amplified in the presence of co-inoculation with a known MHB such as *B. amyloliquefaciens* [17] in addition to an AM fungus. A key question this study aims to address is, can the benefits of the MHB *B. amyloliquefaciens* be enhanced further in the presence of additional AM fungi through co-inoculation? Further, is the benefit realised to a greater degree under different tillage regimes? The current study aims to investigate the nature of the tripartite relationship between selected *Bacilli* spp. rhizobacteria, AM fungi and winter wheat in the context of species applied as inoculants (_[i]_) and the naturally occurring soil borne species (_[s]_) under two tillage regimes, CT and zero tillage (ZT). Microbial inocula are known to affect plant growth, and therefore, the present study is testing the hypothesis that different combinations of inoculants are likely to affect plant growth differently depending on different mutual synergisms between inocula. Indicators of enhanced symbiosis include plant biomass (tiller number and length, root length), root arbuscule count and soil glomalin concentration. The glycoprotein glomalin is produced by AM fungi along their mycelial network where it acts as a structural support molecule [24], functioning as an adhesive glue-like compound [25]. Its presence is indicative of AM fungal growth [2]. Naturally occurring PGPR and AM fungi in the field extracted soils have been determined by qPCR analysis. The impact of combined PGPR and AM fungi inocula has been compared with three baselines: (1) control (no inoculation), (2) the best performing single PGPR inoculum (PGPR_[i]_) and (3) inoculation with *R. intraradices* only (AMF_[i]_). Co-inoculation has been deemed beneficial only where an improvement to (2) and (3) has been identified.

## 2. Materials and Methods

### 2.1. Sample Sites

Soil sampling was conducted at two commercial farms in Hertfordshire (Farm A near Hitchin; Farm B Hatfield), UK, using a Dutch auger to a depth of 10 cm for topsoil removal and immediate addition on-site to a plastic pot (7 cm radius, 9 cm depth) pre-sterilised with 1% angiene^®^ and rinsed in distilled water. The pots plus soils were transported in closed 100 L plastic storage containers. For both farms the nearest climate station was Rothamsted (51.809568 latitude, −0.35632215 longitude; mean annual rainfall 712.3 mm, mean minimum and maximum annual temperature 6.0–13.7 °C) (UK Meteorological Office, 2018). Both farms were commercially managed, with the current management regime undertaken for a period of at least eight years at the time of sampling. Farm A implemented CT, soil inversion with a moldboard plough to a depth of 20 cm. Farm B practiced ZT by direct seed sowing (John Deere^®^ 750A direct drill, Moline, IL, USA). The broad-spectrum herbicide glyphosate (360 g L^−1^ active ingredient, 3.0 L ha^−1^, CleanCrop Hoedown^®^, Agrovista UK Ltd., Nottingham, UK) was applied to the ZT wheat crop during seed-bed preparation in September as a weed control measure. According to the methodology of Brown and Wherrett [26], the soil at both farms was sandy loam. Each field in Farm A and B was selected based on the presence of a comparable crop history and type and application rate of supplementary crop nutrients. Both farms used RB209 [27] fertiliser recommendations for winter wheat grown on a sandy loam soil.

### 2.2. Rhizobacteria and AM Fungi Inoculum

Following the method described in Wilkes et al. [17], rhizobacteria were derived from a commercially available (Tribus^®^ original, Impello Biosciences, Fort Collins, Colorado, USA) mixture consisting of three Gram-positive Bacilli species: *B. subtilis*, *B. pumilis* and *B. amyloliquefaciens* at a combined cfu count of 10 billion mL^−1^. The identity of each of the three species was confirmed in the laboratory by API^®^ (bioMerieux^®^ Ltd., Marcy l’Etoile, ARA, France) colour guides. Each *Bacilli* spp. was extracted from an isolated colony of pure growth samples obtained from serial dilutions and streak plate purification before analysis with API^®^ 50 CH (bioMerieux^®^ Ltd., Marcy l’Etoile, ARA, France) in triplicate and confirmation in reference to the API^®^ database. The AM fungal inoculum was cultured to a total mass of 5 g in 200 mL nutrient broth for 1 week and homogenised with an electric blender for 1 min. The AM fungal inoculum was obtained from wet sieving through a series of 40, 35 and 28 µm mesh sizes in succession. Collected spores were divided into morphology under stereomicroscope (Apex^®^ stereomicroscope, Wiltshire, UK) at a magnification of ×50 following the protocol described by Sun et al. [28]. Spores identified as AM fungal were grown in a 250 mL conical flask of Czapek Dox broth with surface sterilised wheat roots attached to the side of the flask at 15 °C for 1 week. Wheat roots were surface sterilised by submerging in absolute ethanol or 30 s, followed by soaking in sodium hypochlorite (NaClO) for 30 min, with a further 20 s wash with absolute ethanol to remove residual sodium hypochlorite. Finally, the root tissues were rinsed in distilled water. Eurofins Scientific^®^ (Wolverhampton, West Midlands, UK) confirmed the species identity as *R. intraradices*.

### 2.3. Impact of Rhizobacteria on AM Fungi Root Arbuscule Number and Winter Wheat Growth

Winter wheat (variety Zulu) plants (n = 160 total: 5 plants per treatment, 8 treatments, 2 tillage regimes, spatially arranged in a Latin square design, 2 sample times) were inoculated with a single rhizobacteria species (*B. subtilis*, *B. pumilis*, *B. amyloliquefaciens*), one species of AM fungi (*R. intraradices*) and three combination inoculants of one rhizobacterial species plus one AM fungi. Each *Bacilli* spp. rhizobacteria was suspended in phosphate buffer solution (PBS) following centrifugation at 100× *g* for 1 min to form a pellet of 10,000 cfu. Each plant was grown individually in a plastic pot (7 cm radius, 9 cm depth) under glasshouse conditions (20 °C, 37% relative humidity, 15,260 lux) in soil collected from one of two tillage treatments, CT or ZT. The isolated 10,000 cfu *Bacilli* spp. rhizobacteria were applied as a suspension at a rate of 2 mL to the soil at the base of each winter wheat plant replicate to simulate inoculation in a field grown crop. A single 2 mL application was made to each plant every seven days for 30 weeks, i.e., 30 applications in total. Soil samples (3 g total) were taken weekly and assessed for nitrogen (N), phosphate (P_2_O_5_) and potassium (K) nutrient content. The wheat plants were destructively sampled at weeks 15 (GS30 [29]) and 30 (GS39 [29]) and the root length measured before staining using the protocol developed by Wilkes et al. [22] to obtain AM fungi arbuscule counts. Samples were fixed in a 10% formaldehyde (CH_2_O), 50% ethyl alcohol (CH_3_CH_2_OH), 5% acetic acid (CH_3_COOH) *v/v* (FAA) solution for 24 h; autoclaved in deionised water and incubated at 60 °C in 5% *v/v* hydrochloric acid (HCl) for 1 h. Roots were divided into 5 × 1 cm sections and stained with 10% *v/v* Sheaffer^®^ Blue ink in 5% glacial acetic acid for three minutes, subject to root squash with root tissues sealed between microscope slides with clear nail varnish (nitrocellulose [C_6_H_7_(NO_2_)3O_5_] dissolved in ethyl acetate [C_4_H_8_O_2_]) and viewed under a light microscope at ×100 magnification. Finally, the number of tillers, tiller length and root length were recorded for each replicate of winter wheat.

### 2.4. Impact of Rhizobacteria on Soil Glomalin

Soil glomalin, used as an indicator of AM fungal growth, was quantified from 160 × 100 g replicates of field sampled soil for the two tillage treatments, respectively. Soil (16 kg) was extracted from within 10 cm of the soil surface in the centre of each field post wheat harvest during August 2019. Soils were placed into plastic plant pots (7 cm diameter × 9 cm height) and placed into plastic, non-draining trays with 2 L water applied once per week grown under controlled glasshouse conditions (20 °C, 37% humidity, 15,260 lux). A total volume of 2 mL, 10,000 cfu of each of the three isolated rhizobacteria *Bacilli* spp. (*B. subtilis*, *B. pumilis* or *B. amyloliquefaciens*) or *R. intraradices* AM fungal inoculum (5 g mycelium in 100 mL phosphate buffer solution, 2 mL applied per plant) as well as combination inoculations of single rhizobacteria and AM fungi were applied to the soil surface of each respective replicate every seven days for 30 weeks. Control samples received an application of sterile phosphate buffer solution. Glomalin extraction from each soil sample followed a modified methodology from Wright and Upadhyaya [30] to ascertain total soil glomalin (TSG). In brief, 1 g of soil was suspended in 8 mL of 50 mM trisodium citrate dihydrate and maintained at 121 °C/15 psi in an autoclave for 60 min. Soils were then centrifuged at 1000× *g* for two minutes to remove suspended soil particles. The supernatant was further centrifuged at 6800× *g* for 15 min to remove impurities within the sample of which 1 mL was then extracted for use in the Bradford Protein Assay (Coomassie Protein Assay Reagent, Thermo Fisher Scientific^®^, Loughborough, Leicestershire, UK) (Kruger, 2009) using a Cecil 1021^®^ photo-spectrometer (Cambridge, UK) at an absorbance of 595 nm.

### 2.5. Soil Nutrient Testing

Nitrogen: 1 g of dried field extracted soil and 0.04 g calcium sulphate (CaSO_4_) agitated together for 30 min in a total volume of 5 mL de-ionised water then filtered with Whatman No.1^®^ filter paper. 1 mL of filtrate was analysed using HACH^®^ LCK 339 nitrate testing kits.

Phosphate: 1 g of dried soils added to 20 mL phosphate extraction solution (in 500 mL distilled water, 3 g ammonium sulphate (NH_4_)_2_SO_4_ and 20 mL concentrated sulfuric acid H_2_SO_4_) and agitated for 30 min then filtered (Whatman No.1^®^ filter paper). A volume of 10 mL of filtrate was added to 20 mL de-ionised water and 2 mL molybdate solution (in 500 mL distilled water, 10 g ammonium molybdate (NH_4_)6Mo_7_O_24_ and 240 mL concentrated H_2_SO_4_) and one spatula of ascorbic acid in excess, before heating to boiling point. After the samples had cooled to room temperature, photospectrometry (model: Cecil^®^ 1021) at a wavelength of 650 nm absorbance was conducted, using deionized water as a reference. A standard curve was constructed using known phosphate concentrations.

Potassium: 1 g of dried soil added to 20 mL extraction solution (in 1 L distilled water, 15.4 g calcium lactate (C_6_H_10_CaO_6_), 17.9 mL glacial acetic acid CH_3_COOH) and agitated for 60 s to produce a soil suspension. After allowing the suspension to settle for 20 min, the agitation and settlement stages were repeated a further two times before filtering through Whatman No.1^®^ filter paper. If the filtrate was cloudy, or contained soil debris, a single spatula of activated carbon was added prior to agitation and re-filtering. The filtrate was adjusted to between pH 5 and 7 with 32% sodium hydroxide (NaOH) solution. A total of 30 mL of 2:1 EDTA disodium salt (C_10_H_14_N_2_Na_2_O_8_), 20% formaldehyde (CH_2_O) respectively and 1 mL sodium tetraphenylborate (C_6_H_5_)4BNa (5% *w*/*v*) were added per 3 mL of pH adjusted filtrate then mixed in a Vortex-Genie^®^ 2 (Scientific Industries SI^®^, Bohemia, NY, USA) at 3200 rpm. The extracted solution served as a reference during the photospectrometry (model: Cecil^®^ 1021) analysis at a wavelength of 690 nm absorbance. Spectrograph readings were compared to a constructed standard curve of known concentrations.

### 2.6. Molecular Quantification of AM Fungi and Rhizobacteria Species with qPCR

Microorganism deoxyribonucleic acid (DNA) was extracted from 0.2 g of glasshouse grown wheat plant rhizosphere soil (soil adhered to the plant root) with a soil DNA purification kit (ThermoFisher^®^, Waltham, MA, USA). Nucleic acid extraction was confirmed using the NanoDrop^®^ software program. The extracted DNA was mixed with Environmental Master Mix 2.0 (ThermoFisher^®^) and custom TaqMan assays (ThermoFisher^®^) produced for *B. subtilis* (Assay ID: APGZJG4), *B. pumilis* (Assay ID: APT2DHH), *B. amyloliquefaciens* (Assay ID: APWCZNA) and *R. intraradices* (Assay ID: APYMNUJ). Each sample was extracted in multiples of four, one for each species to be identified, to produce 96 samples in total (CT and ZT, eight treatments each, 15 and 30 weeks) for each custom TaqMan^®^ assay, run in triplicate for each species. An Antigen thermocycler performed the qPCR analysis with thermo cycles at 95 °C for 10 min followed by a 60-cycle sequence of 15 s at 95 °C, 1 min at 60 °C and 1 min at 72 °C. The amount of fluorescence was recorded and compared to a constructed logarithmic standard curve of copy numbers.

### 2.7. Statistical Analysis

Statistical analyses were conducted using the R commander^®^ (Hamilton, ON, Canada) software package. The mean and standard error was calculated for each set of sample data. A multi-variate ANOVA tested for differences between inoculum treatments and tillage regime at the same time of sampling (week 15 or week 30). A single factor ANOVA tested for differences between inoculum treatments within the same tillage regime. Where significant differences were identified, paired two-tail *t*-tests of equal variance were employed for post hoc null hypothesis testing within the same tillage treatment. Further paired two-tail *t*-tests of unequal variance were applied to sample analysis between tillage treatments (the soil disturbance was not equal). Statistical significance was determined by *p* values ≤ 0.05.

## 3. Results

**Figure 1:**
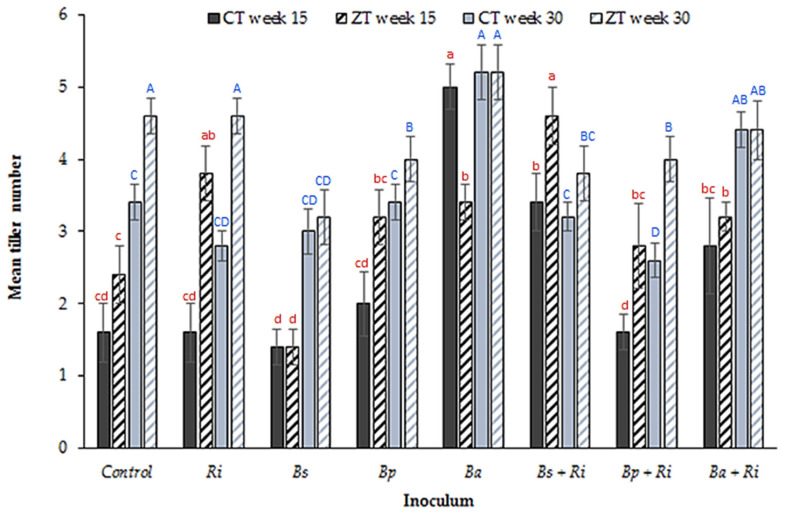
Mean (n = 80 plants per tillage treatment) number of tillers from glasshouse grown winter wheat in CT and ZT extracted field soils at weeks 15 and 30 post germination for seven inoculant combinations (where Ri: *R. intraradices*, Bs: *B. subtilis*, Bp: *B. pumilis*, Ba: *B. amyloliquefaciens*) and a control. Different letters denote significant differences between treatments (inoculum and tillage regime) for the same sampling week (week 15 red lower case, week 30 blue upper case). Error bars = ±one standard error of the mean.

The testing of key soil nutrients (NPK) of soils in the pots revealed no statistically significant difference between the control non-inoculated soils and soil inoculated with either single *Bacilli* spp. and/or *R. intraradices* (*p* = 0.79, degrees of freedom (df): 7, 103, F value: 13.88, single factor ANOVA). A multi-way ANOVA comparing inoculant and tillage treatment at the same sampling time indicated a significant difference between treatments for tiller number, a proxy indicator of plant growth, in winter wheat (*p* < 0.00001, df: 7, 71271, F value: 20.70). Numbers were greater in ZT soils during weeks 15 and 30 (Figure 1), with the exception of single *B. amyloliquefaciens* inoculations in CT soils at week 15 (*p* < 0.00001, df: 7, 32, F value: 9.38, single factor ANOVA). A post hoc 2 tailed paired *t*-test of equal variance conducted post ANOVA showed *B. amyloliquefaciens* inoculum increased overall tiller significantly in both the CT and ZT treatments (*p* < 0.0001, df: 8, t.stat: −3.89, paired equal variance *t*-test) except the ZT week 15.

**Figure 2:**
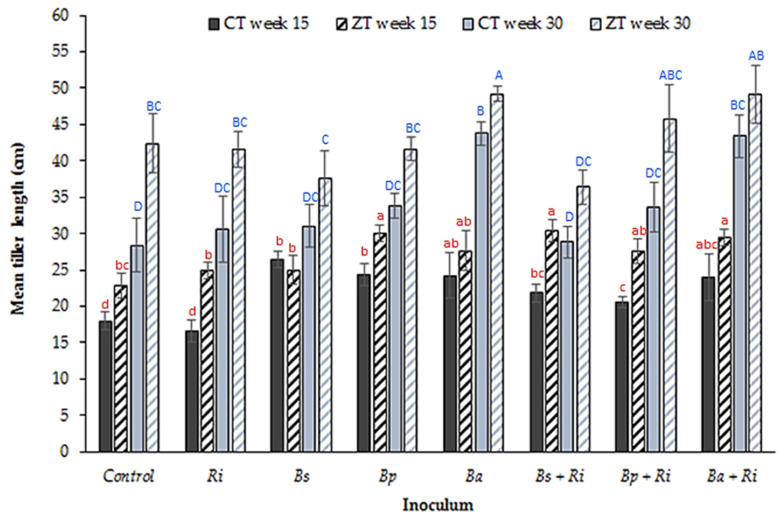
Mean (n = 80 plants per tillage treatment) tiller length (cm) from glasshouse grown winter wheat in CT and ZT extracted field soils at weeks 15 and 30 post germination for seven inoculant combinations (where Ri: *R. intraradices*, Bs: *B. subtilis*, Bp: *B. pumilis*, Ba: *B. amyloliquefaciens*) and a control. Different letters denote significant differences between treatments (inoculum and tillage regime) for the same sampling week (week 15 red lower case, week 30 blue upper case). Error bars = ±one standard error of the mean.

*Bacillus amyloliquefaciens* was also key in enhancing tiller length. Inoculation overall significantly increased tiller length in both CT (*p* = 0.03, df: 7, 32, F value: 5.57, single factor ANOVA) and ZT extracted soils (*p* = 0.01, df: 7, 32, F value: 6.57, single factor ANOVA) at week 15 (Figure 2). Three treatments (*B. pumilis* only, AM fungi + *B. subtilis* and AM fungi + *B. amyloliquefaciens* co-inoculations) induced a significant change in ZT at week 15 (*p* < 0.01, df: 8, t.stat: −3.54, paired equal variance *t*-test). In the CT treatment, this was limited to *B. amyloliquefaciens* inoculum alone (*p* < 0.01, df: 8, t.stat: −3.83, paired equal variance *t*-test) at week 30.

Treatments observed to have a significant impact at week 30 include *B. amyloliquefaciens* inoculant and AM fungi + *B. amyloliquefaciens* co-inoculation in CT (*p* < 0.002, df: 7, 32, F value: 4.08, single factor ANOVA) and ZT treatments, although not significant in the latter (*p* = 0.05, df: 7, 32, F value: 2.21, single factor ANOVA).

**Figure 3:**
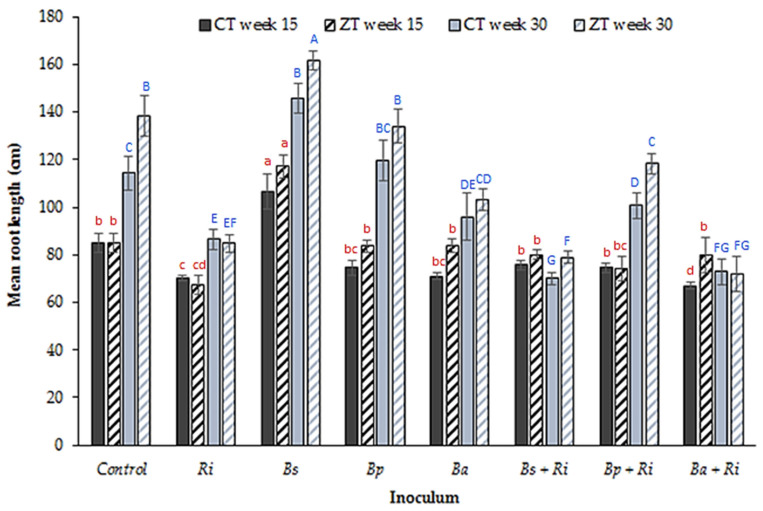
Mean (n = 80 plants per tillage treatment) root length (cm) from glasshouse grown winter wheat in CT and ZT extracted field soils at weeks 15 and 30 post germination for seven inoculant combinations (where Ri: *R. intraradices*, Bs: *B. subtilis*, Bp: *B. pumilis*, Ba: *B. amyloliquefaciens*) and a control. Different letters denote significant differences between treatments (inoculum and tillage regime) for the same sampling week (week 15 red lower case, week 30 blue upper case). Error bars = ±one standard error of the mean.

Root length (Figure 3) increased in ZT soils (*p* < 0.00001, df: 7, 32, F value: 10.86, single factor ANOVA) relative to CT soils (*p* < 0.00001, df: 7, 32, F value: 13.88, single factor ANOVA). *Bacillus subtilis* was the only inoculant to increase root length significantly (*p* < 0.01, df: 8, t.stat: −5.01, paired equal variance *t*-test), but this was only when applied singularly, not as a co-inoculant with AM fungi. The application of AM fungi decreased overall root length (*p* < 0.01, df: 8, t.stat: 2.98, paired equal variance *t*-test) relative to the control treatment in both the CT and ZT treatments. Significant decreases were also evident for *B. amyloliquefaciens* inoculations alone and for the AM fungi + *B. pumilis* and AM fungi + *B. amyloliquefaciens* co-inoculations (*p* < 0.01, df: 8, t.stat: 3.21, paired equal variance *t*-test) in CT soils.

**Figure 4:**
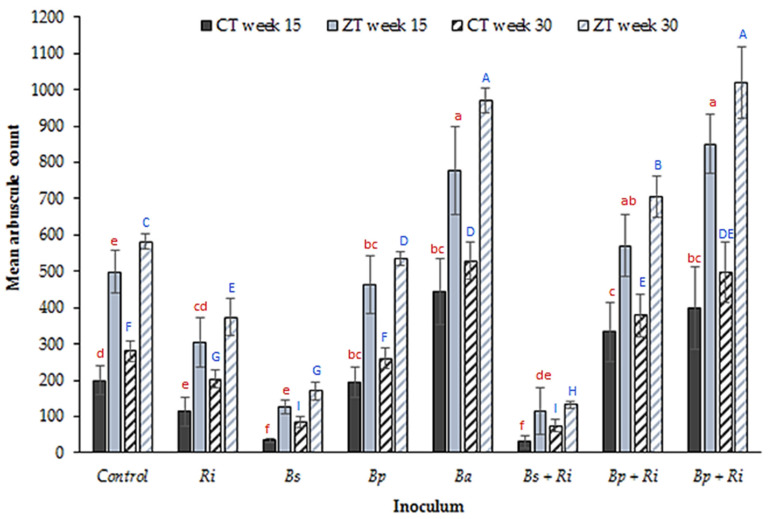
Mean (n = 80 plants per tillage treatment) root arbuscule count per 1 cm root section from glasshouse grown winter wheat in CT and ZT extracted field soils at weeks 15 and 30 post germination for seven inoculant combinations (where Ri: *R. intraradices*, Bs: *B. subtilis*, Bp: *B. pumilis*, Ba: *B. amyloliquefaciens*) and a control. Different letters denote significant differences between treatments (inoculum and tillage regime) for the same sampling week (week 15 red lower case, week 30 blue upper case). Error bars = ±one standard error of the mean.

**Figure 5:**
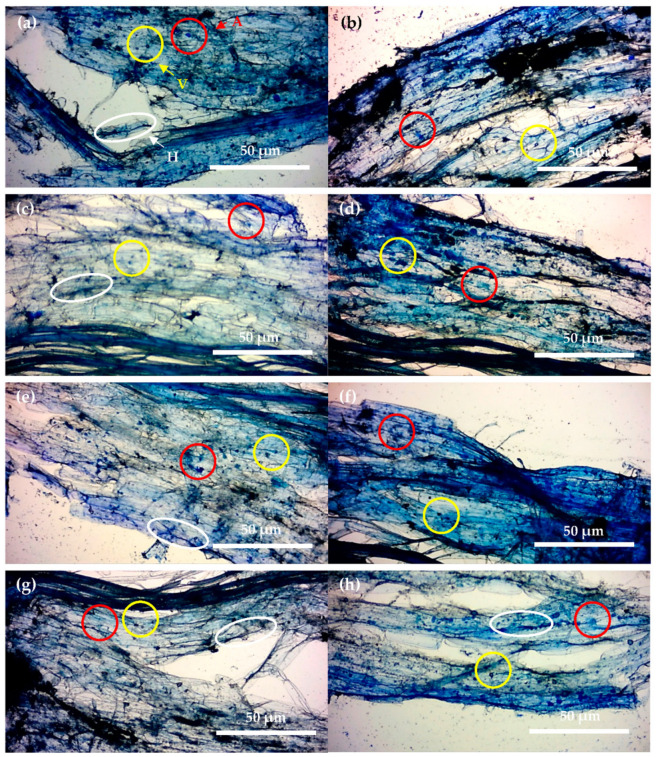
Images of Sheaffer^®^ blue stained root sections from winter wheat grown in collected ZT soils at 15 weeks of growth. A—arbuscule, V—vesicle, H—intra-radiating hyphae. (**a**) control, (**b**) *R. intraradices*, (**c**) *B. subtilis*, (**d**) *B. amyloliquefaciens*, (**e**) *B. pumilis*, (**f**) *R. intraradices* + *B. subtilis*, (**g**) *R. intraradices* + *B. amyloliquefaciens*, (**h**) *R. intraradices* + *B. pumilis*, taken using an Apex^®^ microscope at a total magnification of ×100 and a Bresser^®^ HD microscope camera.

Root arbuscule count (Figure 4), an indicator of wheat plant AM fungal symbiosis, was significantly higher in the ZT compared to the CT treatment during both weeks 15 and 30 (*p* = 0.002, df: 7, t.stat: 4.20, unequal variance paired *t*-test). Arbuscule count declined significantly relative to the control in both the CT and ZT treatments in response to inoculation with *R. intraradices* (*p* = 0.03, df: 8, t.stat: 2.15, equal variance paired *t*-test); however, *R. intraradices* + *B. amyloliquefaciens* inoculations increased root arbuscules significantly (*p* = 0.005, df: 7, t.stat: 3.48, equal variance paired *t*-test). There was a further significant difference in arbuscule count between the *R. intraradices* and *R. intraradices* + *B. amyloliquefaciens* inoculants (*p* < 0.001, df: 8, t.stat: 5.11, equal variance paired *t*-test) in both tillage types at both sampling weeks suggesting that further additive increases symbiosis resulted from co-inoculation. However, no significant difference was present between *R. intraradices* + *B. amyloliquefaciens* tillage types at both week 15 and week 30 sampling (*p* = 0.11, df: 8, t.stat: 1.33, unequal variance paired *t*-test). Micrographs of stained root samples from each inoculant treatment illustrating arbuscules and other structures (vesicles and intra-radiating hyphae) are shown in Figure 5.

**Figure 6:**
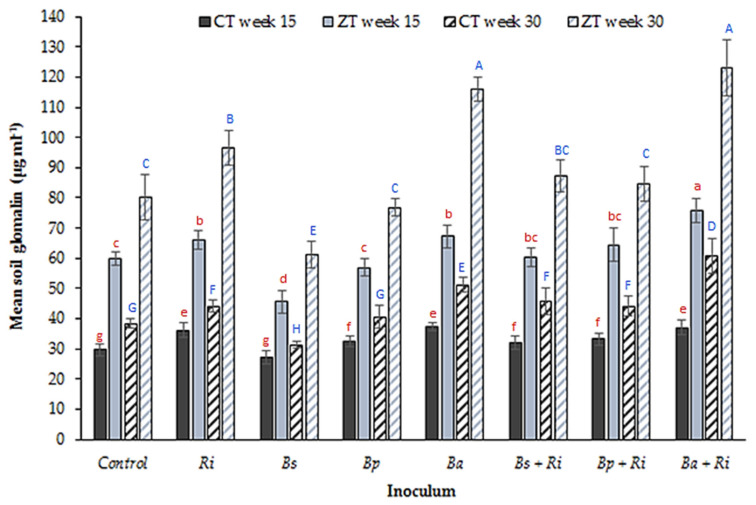
Mean (n = 80 soil samples per tillage treatment) soil glomalin (µg mL^−1^) from glasshouse grown winter wheat in CT and ZT extracted field soils at weeks 15 and 30 post germination for seven inoculant combinations (where Ri: *R. intraradices*, Bs: *B. subtilis*, Bp: *B. pumilis*, Ba: *B. amyloliquefaciens*) and a control. Different letters denote significant differences between treatments (inoculum and tillage regime) for the same sampling week (week 15 red lower case, week 30 blue upper case). Error bars = ±one standard error of the mean.

Soil glomalin concentration (Figure 6) was significantly higher in the ZT treatment compared to CT during week 15 (*p* < 0.0001, df: 8, t.stat: 10.24, unequal variance paired *t*-test) and week 30 (*p* < 0.0001, df: 8, t.stat: 10.24, unequal variance paired *t*-test) which, as an indicator of AM fungal biomass, suggests soil conditions more conducive to enhanced AM fungal growth. A significant difference was evident between inoculant treatments at week 15 in the ZT soils only (*p* < 0.001, df: 7, 32, F value: 12.57, single factor ANOVA). A post hoc 2 tailed paired t-test of equal variance identified that soils inoculated with *B. amyloliquefaciens* (*p* < 0.001, df: 8, t.stat: 4.51, equal variance paired *t*-test) and *R. intraradices* + *B. amyloliquefaciens* (*p* < 0.001, df: 8, t.stat: 4.51, equal variance paired *t*-test) had significantly greater soil glomalin compared to the control treatment in the ZT extracted soils. At week 30, glomalin increased in both the CT (*p* < 0.00001, df: 7, 32, F value: 16.33, single factor ANOVA) and ZT (*p* < < 0.0001, df: 7, 32, F value: 5.09, single factor ANOVA) treatments, suggesting a greater lag effect in the CT treatment, possibly due to the lower baseline glomalin concentration in the control treatment. Observations were comparable to the other variables analysed in that although significantly greater than the control treatment, co-inoculation using a single PGPR with *R. intraradices* did not enhance soil glomalin levels above that of a single species inoculant. There was no significant difference between *B. amyloliquefaciens* and *R. intraradices* + *B. amyloliquefaciens* (*p* = 026, df: 7, t.stat: 0.70, equal variance paired *t*-test).

The qPCR analysis of soil extracted microorganism DNA from the CT and ZT field locations (Table 1) identified the presence of only one PGPR inoculant species, *B. amyloliquefaciens.* Further, the presence of this species in the control treatments suggests the presence of this species as a naturally occurring population in the extracted field soils used in the experiment. The qPCR copy numbers were greatest in the soils of the CT treatment, implying soil conditions and the associated CT management protocols were, in this case, more suitable to the ecological requirements of *B. amyloliquefaciens*.

## 4. Discussion

A study of the impact of select *Bacilli* spp. rhizobacteria and the AM fungi *R. intraradices* as both single and combined inoculations within field extracted arable soils of two tillage treatments, CT and ZT, at crop growth stages GS30 and GS39 [29] has been conducted. The treatments contain naturally occurring soil borne species (PGPR_[s]_ and AMF_[s]_) and species introduced via inoculation (PGPR_[i]_ and AMF_[i]_), being able to uphold the tested hypothesis with noticeable and measurable increases to crop tillers and root length. Under an ideal scenario, the following sequence of events highlighted as a multipartite association between the host plant, AM fungi and bacteria by [31] would occur. The solubilizing of phosphorous combined with the fixing of nitrogen to improve nutrients availability in the soil which are then absorbed and translocated more efficiently to the winter wheat plant by the extraradical hyphae of an AM fungi [31], in this case *R. intraradices*. Simultaneously, siderophores produced by PGPR suppress soil rhizosphere plant pathogens while the auxins synthesised by PGPR promote plant growth [31]. A component not mentioned by [31] is the MHB role of the PGPR to facilitate root colonisation by AM fungi which further enhances nutrient assimilation and plant growth. The components of this multipartite association are, according to the literature, potentially present in the PGPR and AM fungi inoculations used here. A further factor is the enhancement of naturally occurring soil dwelling microorganisms (PGPR_[s]_ or AMF_[s]_) through modification to soil management, in this case tillage. With reference to specific species, it might include *B. amyloliquefaciens* to solubilise phosphorous, *B subtilis* to suppress plant pathogens and *R. intraradices* to improve resource translocation efficiency. Although beneficial relative to the control plants, co-inoculation with PGPR_[i]_ and AMF_[i]_ did not improve plant leaf biomass, root arbuscule count or soil glomalin compared to a single inoculation. There are evidently a number of different mechanisms in operation here, the ability of the AM fungi and PGPR to colonise the plant root, the competitive ability for resources with other microorganisms and the disease suppression capabilities that translate not just to plant pathogens but also non-pathogenic species and those that offer potential plant growth benefits.

*Bacillus amyloliquefaciens* was the only PGPR to be present in the soil (PGPR_[s]_) of the non-inoculated control treatments but limited to the CT sampled location and extracted soils, suggesting the conditions created by tillage are more suitable for this PGPR species. Two key mechanisms appear to be in operation. Tillage is detrimental to AMF_[s]_ that facilitate nutrient transfer to the crop plant and enhance growth, due to the destruction of hyphal networks [2,20,32]. The aerobic soil conditions created by soil inversion, however, benefit the gram positive PGPR_[s]_ *B. amyloliquefaciens* [33], noted to benefit plant growth at earlier growth stages by [17] and the later growth stages in this study. Wilkes et al. [34] also report the detrimental impact of the herbicide glyphosate on AM fungi although they do not quantify the impact on PGPR. Glyphosate inhibits critical enzyme function within plants, namely the enzyme 5-endolpyruvylshikimate-3-phosphate (EPSP) synthase via the Shikimate pathway [35,36]. Both the Shikimate pathway and EPSP synthase are components of most fungi and bacteria [37,38]. A number of authors suggest that the impact of glyphosate on PGPR is also negative due to this, which potentially explains the absence of *B. amyloliquefaciens* in the ZT extracted control soils. Escobar Ortega [39] report that microbial communities associated with the rhizosphere, i.e., both bacteria and AM fungi, were inhibited by aminomethylphosphonic acid (AMPA), a metabolite formed during the degradation of glyphosate. *Bacillus subtilis* is named specifically as being inhibited by AMPA [40]. Further, because of its persistence in soils [41], it accumulates within the soil rhizosphere [42,43]. The inhibitory effect at standard field application rates may, therefore, be prolonged, as noted in corn and soybean crop [44]. Rhizosphere microorganisms are reported to tolerate glyphosate at concentrations up to 250 mg mL^−1^ [45]. Wilkes et al. [4] found that glyphosate applied at a rate equivalent to 10 mg mL^−1^ inhibited AM fungi growth. Further, Wijekoon and Yapa [46] hypothesise that the negative impact of glyphosate on PGPR reduces the ability of such species to compete with, and reduce infection by, plant pathogens. Pathogenic species tend to be gram negative and more suited to anaerobic soil conditions. The non-inversion of the soil and greater prevalence of anaerobic conditions this creates, in combination with an inhibitory effect induced by AMPA, decreases the suitability of the soil environment to PGPR such as *B. amyloliquefaciens*. This could explain in part the lower *B. amyloliquefaciens* in the ZT treatment compared to CT.

There are two important questions to address. Firstly, was *B. amyloliquefaciens* beneficial as an inoculant because it was the main naturally occurring species identified in CT soils? which in theory then follows that the symbiotic relationship with the crop plant is well developed and established. Supplementary application of this species is therefore beneficial, especially in ZT systems where the abundance may be lower, but that species absence is potentially due to management suppressing populations, not that this species is absent as a naturally occurring species otherwise. The survival time of inoculant species tends to be low [47]. Inoculation with a species found to be naturally occurring may however permit the inoculant to persist for longer. Co-inoculation of native *Geastrum coronatum* + PGPR was of greater benefit than inoculation with a non-native species, *G. intraradices* [48] leading to Bashan and Levanony [49] to conclude that the change in the soil microbiome due to inoculation was at best only temporary. Secondly, in addition to the relationship with the host plant, did it have a beneficial and well-established relationship with the naturally occurring AMF_[s]_ in the soil, i.e., does not suppress AM fungi growth? Soil glomalin in the control samples suggests the presence of naturally occurring AM fungi; however, it is possible that the abundance was low such that the soil DNA extraction was unable to isolate it sufficiently for the qPCR analysis. In response to the first question, the potential for a well-developed symbiotic relationship with winter wheat is high since the rotation in the fields from where the soil was extracted for use in the experiments had included winter wheat for many years. The intrinsic ecological relationship that exists between the host plant and AM fungi in the rhizosphere, and the potential for this to extend to PGPR also is reported by McGonigle and Fitter [50]. Root exudates provide an ecological niche for soil microorganisms [51], with organic or amino acids and sugars of particular importance [52]. An interesting observation is that a proportion of these plant root exudates may exhibit antimicrobial properties and therefore permit a select number of species, including PGPR, to occupy the ecological niche within the rhizosphere of that particular plant species [53,54]. In this case, the naturally occurring PGPR need to be tolerant of the winter wheat root exudates and be able to tolerate the soil conditions created by either soil inversion or ZT. For those species capable of exploiting these conditions, a beneficial relationship with the plant species that provides these resources, one that will enhance growth and simultaneous release of root exudates, is beneficial to the PGPR. The specificity of the host—PGPR interaction is reported for soybean [55] and note that root exudates attract *B. amyloliquefaciens* specifically. This interaction is, however, further evident at the intraspecies level. While *B. amyloliquefaciens* is present as a naturally occurring species (PGPR_[s]_) in CT soils, inoculation with *B. amyloliquefaciens* (PGPR_[i]_) may not necessarily confer the same benefits, although in this study it did appear to be the case. Reva [55] found that two different strains of *B. amyloliquefaciens* had different root colonising capabilities. Strain UCMB-5017 was inhibited and killed by bactericidal plant root exudates. In contrast strain UCMB-5113 secreted a protective layer and additional surface structures that attached it to the root surface. This interaction appears to be host plant and PGPR strain specific. The *B. amyloliquefaciens* strains UCMB-5036 and UCM B-5044 that were effective colonisers of cotton roots were unable to colonise barley or oilseed rape plant roots [55]. Two key questions arise from this. Is *B. amyloliquefaciens* specific to winter wheat? And if so, is this further specific to the Zulu variety of wheat? No specific *B. amyloliquefaciens* winter wheat relationship is currently reported in literature. The abundance of this species in fields in which a winter wheat monoculture has dominated the rotation for several decades would suggest that some form of beneficial host specific relationship exists, at least for the given climatic and soil conditions for the current study.

The second question to address, given the presence of naturally occurring soil borne *B. amyloliquefaciens* (PGPR_[s]_) in the control CT extracted soils but no presence of *B. pumilis* or *B. subtilis* is, is there a beneficial and established relationship with the naturally occurring AMF_[s]_ in the soil? Further, is this beneficial PGPR_[s]_–AMF_[s]_ relationship for *B. amyloliquefaciens* also present for the PGPR_[i]_ species, i.e., a positive relationship observed for PGPR_[i]_–AMF_[s]_ inoculated as *B. amyloliquefaciens* only, but not *B. pumilis* or *B. subtilis*. It offers a potential explanation as to why inoculation with *B. amyloliquefaciens* was the only species to confer a benefit. An important point is made by Dunstan et al. [56]. They note that the nature of the interaction between PGPR and AM fungi is species specific, i.e., a particular PGPR species may interact differently with different AM fungi species. In this case, although the relationship between *B. amyloliquefaciens* PGPR_[i]_ and AMF_[s]_ was positive, the relationship with inoculated *R. intraradices* (AMF_[i]_) was neutral as indicated by the number of tillers (Figure 1), the mean arbuscule count (Figure 4) and the soil glomalin concentration (Figure 6). It is noted by Andrade et al. [57] that the hyphae of AM fungi may decrease in length in the presence of PGPR that are not the preferred host, creating an antagonistic relationship. This may explain the decline in tiller number and length as well as arbuscule count noted for inoculation with *B. subtilis*. A decrease in hyphal length reduces the capacity of the AM fungi to acquire nutrients for the host plant [57], decreasing the efficiency of the symbiotic relationship. Wilkes et al. [17] observe an increase in root length indicative of the need by the plant to assimilate nutrients mainly via its own rooting system rather than through the increased acquisition efficiency provided by the AM fungi hyphae. This relationship may be site specific, with different PGPR interacting positively or negatively with a given species of AM fungi depending on the profile of the naturally occurring soil microorganisms. In reference to [56] this represents the rhizosphere of the wheat plant and the associated PGPR_[s]_ and AMF_[s]_ present for the locations sampled in this study only. It would not necessarily apply to other crops and fields where different species of AMF_[s]_ dominate, or indeed where the species of PGPR_[s]_ and the nature of the established symbiotic relationship potentially differ. The application of *B. subtilis*, an apparent antagonist in this study, may actually confer benefits if this is the main species of PGPR_[s]_ for a given location and has established a symbiotic relationship with the AMF_[s]_ [31,56]. The inoculation of agronomically important plant species therefore as PGPR_[i]_ and AMF_[i]_ would, based in this assertion, require the evaluation of the existing PGPR_[s]_ and AMF_[s]_ communities and the adaptation of the inoculated species accordingly.

If *B. amyloliquefaciens* is a beneficial naturally occurring species (PGPR_[s]_) capable of exploiting the winter wheat rhizosphere, another important factor to consider is the suppressive effect of this species on other potentially pathogenic bacteria. The suppression of pathogenic microorganisms may occur through two mechanisms: (1) competition for the same resource or (2) biocontrol. The competition for resources between microorganisms is highlighted by [51], such that a pathogenic species is disadvantaged in the competitive interaction and growth is hindered, is one mechanism whereby the host plant receives protection. Soluble iron is a micronutrient of importance to microorganisms in soils due to its presence in growth limiting quantities [31]. In microorganisms, the assimilation of iron occurs via iron transporters or siderophores [58] that vary in their efficiency, with pathogenic bacteria typically more vulnerable to iron deprivation due to lower assimilation efficiency [59]. A second mechanism is the production of secondary metabolites by a non-pathogenic species that have toxicity toward a pathogen thereby suppressing growth and infection [60,61]. Co-inoculation with PGPR and AM fungi may have potential benefits through the realisation of multiple positive interactions; however, because each interaction is unique, the choice of inoculant PGPR and AM fungi species requires careful consideration. Critically, the two species must be ecologically compatible. This compatibility may be influenced by the nature of the rhizosphere for a given location, i.e., the composite species of PGPR_[s]_ and AMF_[s]_ which will impact the type of interaction observed in response to the application of PGPR_[i]_ and AMF_[i]_. Some species that synthesise biosurfactants, for example, *B. subtilis*, are described as opportunistic pathogens [62]. That is, a particular PGPR is not a pathogen normally but in the presence of other specific PGPR or AM fungi species (i.e., a potential co-inoculant or an inoculant in the presence of a naturally occurring species), pathogenic activity is activated [63]. Co-inoculation under such circumstances would therefore exhibit a negative impact, which offers a potential explanation for the relationship observed in this study for *B. subtilis* and *R. intraradices* inoculation despite *B. subtilis* being reported as conferring benefits in other studies. Similarly, Jäderlund et al. [64] report an increase in the dry weight of wheat plants infested with the plant pathogen *Microdochium nivale*, when inoculated with either *G. intraradices* or co-inoculation of *G. intraradices* + *P. fluorescens* but that this beneficial impact was not observed for co-inoculation of *G. intraradices* + *P. brasilensis*. The former two inoculants conferred a pathogen suppressing effect; the latter did not. In this study, *B. subtilis* inoculant appeared to be antagonistic for the given host plant species and AMF_[i]_ *R. intraradices*. However, according to Chen et al. [65], *B. amyloliquefaciens* (as strain FZB42) synthesises multiple antibacterial and antifungal chemicals. However, in this study, *B. amyloliquefaciens* was not detrimental to the wheat rhizosphere or the co-inoculant *R. intraradices*. In addition to a lack of ecological compatibility between species, there is the influence of the host plant and the associated root exudates, which may be altered under different environmental conditions.

If *B. amyloliquefaciens* is beneficial because it is naturally occurring and not antagonistic to the soil rhizosphere for the sampled location, why does co-inoculation with *R. intraradices* not present further benefits to plant growth? In theory, co-inoculation would be expected to benefit both the CT and the ZT treatment, with the former benefitting from supplementary AM fungi and the latter *B. amyloliquefaciens*. This is not obvious. In their study of two known symbionts, *B. pumilis* and the AM fungi *G. intraradices,* Reference [50] found that both species increased plant growth when applied in isolation, but that there was no impact on host plant growth when the two were applied in combination. Medina et al. [47] also note that the co-inoculation of *Bacilli* spp. and AM fungi conferred intermittent benefit to shoot biomass increase in comparison to plants inoculated with single species separately. They concluded that the AM fungi mycorrhizae enhanced nitrogen uptake rather than phosphate; however, neither nutrient was limited in this case. Tahmatsidou et al. [66] also found that for the suppression of the disease of strawberry, *Verticillium* wilt, inoculation with both a PGPR and AM fungi did not improve disease suppression, i.e., it had a ‘*non-additive*’ response compared to inoculation with a single species, despite both the PGPR_[i]_ and AMF_[i]_ having demonstrated disease suppression when applied in isolation. The authors highlighted the need to identify species and strains that when applied in combination were cumulative rather than neutral or antagonistic. The PGPR_[i]_ applied with the AMF_[i]_, *R. intraradices* in this case, do not appear to satisfy the former criteria, rather they express a neutral or an antagonistic relationship. As discussed earlier, the nature of the relationship may be due to the species-specific composition of the soils under evaluation, and this would then need to be determined on a farm or field by field basis in order to derive appropriate PGPR species mixtures to function as biofertilizers.

Gamalero et al. [67] identify an interesting dynamic in the interaction between PGPR and the plant itself. They report that while a synergistic relationship existed between *Bacillus subtilis* and *Glomus etunicatum* under normal field conditions, when the plant was under environmental stress the relationship was reversed. A shift in the chemical nature of root exudates had decreased or removed the synergistic relationship. The benefit of inoculation and the positive interaction between naturally occurring PGPR and AM fungi and the inoculant species is then potentially realised in full when the target plant is not subject to stressful conditions. The ‘*additive hypothesis*’ of Bashan and Levanony [49] is that the multiple mechanisms that operate simultaneously or that act on a continuum during the crop life-cycle may become negative when one or more of these mechanisms are rendered inactive as a consequence of environmental stress. This may also include the presence of AMPA in the soil rhizosphere inhibiting one or more of said mechanisms. It may to a degree also result from sub-optimal soil conditions where anaerobic conditions are prevalent. Negative interactions may therefore occur under certain environmental or rotation specific conditions and may shift between the two states both during the same cropping year and between cropping years. Under field conditions in northern Europe, this will be more pronounced during the spring when soils are wetter but will potentially shift in favour of PGPR as the season progresses and the soils dry out. The later crop growth stages in a ZT crop are likely to benefit from both PGPR and an intact AM fungi hyphal network. Further work, for example, utilising small-scale field trials to test this hypothesis, would be beneficial. Evaluating the impact of a reduction in the frequency of soil inoculation as a means to develop a more practical farm management approach would be of further interest.

## 5. Conclusions

Selected species of rhizobacteria and AM fungi applied as single inoculants or co-inoculants with AM fungi to winter wheat grown in soils extracted from different tillage treatments have positive, neutral or negative effects on plant growth. This is due to species specific factors (biocontrol and competitive ability) and whether individual species were present as naturally occurring species within the soil of the sample site. The PGPR *B. amyloliquefaciens* applied singularly increased crop biomass and AM fungal symbiosis. Co-inoculation with *R. intraradices* provided no additional improvement or additive benefit. *Bacillus amyloliquefaciens* was the only PGPR found to be naturally occurring in the field extracted soils. The selection of appropriate inoculant PGPR species based on the naturally occurring rhizosphere species may be critical in order to obtain maximum benefit from inoculation or co-inoculation of PGPR and AM fungi.

## Figures and Tables

**Table 1:** Copy numbers of *B. amyloliquefaciens* qPCR samples taken from winter wheat grown in controlled glasshouse conditions at 15 and 30 weeks in soils taken from zero and conventional tillage sites. No copy numbers of *B.subtilis*, *B. pumilis* or AM fungi (as *R. intraradices*) were detected in any treatment.

Tillage Treatment	Inoculant	Week 15	Week 30
Conventional	Control	10^2.5^	10^2.52^
	*B subtilis*	10^2.52^	10^2.49^
	*B pumilis*	10^2.5^	10^2.52^
	*B amyloliquefaciens*	10^1.5^	10^2.52^
	AM fungi	10^2.5^	10^2.52^
	*B subtilis* + AM fungi	10^2.52^	10^2.52^
	*B pumilis* + AM fungi	10^2.5^	10^2.49^
	*B amyloliquefaciens* + AM fungi	10^2.52^	10^2.52^
Zero	Control	0	0
	*B subtilis*	10^1.5^	10^2.49^
	*B pumilis*	0	10^2.5^
	*B amyloliquefaciens*	0	10^1.5^
	AM fungi	0	10^2.49^
	*B subtilis* + AM fungi	0	10^2.52^
	*B pumilis* + AM fungi	0	10^2.5^
	*B amyloliquefaciens* + AM fungi	0	10^2.5^

## Data Availability

Not applicable.
